# Severe congenital microcephaly with 16p13.11 microdeletion combined with *NDE1* mutation, a case report and literature review

**DOI:** 10.1186/s12881-017-0501-9

**Published:** 2017-12-01

**Authors:** Li Tan, Bo Bi, Peiwei Zhao, Xiaonan Cai, Chunhui Wan, Jianbo Shao, Xuelian He

**Affiliations:** 1 Clinical Research Center, Wuhan Children’s Hospital, Tongji Medical College, Huazhong University of Science & Technology, Wuhan, 430016 China; 2 Department of Rehabilitation, Wuhan Children’s Hospital, Tongji Medical College, Huazhong University of Science & Technology, Wuhan, China; 3 Department of CT/MRI Center, Wuhan Children’s Hospital, Tongji Medical College, Huazhong University of Science & Technology, Wuhan, China

**Keywords:** Microcephaly, *NDE1*, 16p13.11 microdeletion, Agenesis of corpus callosum

## Abstract

**Background:**

Microcephaly is a disorder characterized by severe impairment in brain development, reduced brain and head size. Congenital severe microcephaly is very rare, and *NDE1* deletion and genetic mutations are important contributors.

**Case presentation:**

Single nucleotide polymorphism (SNP) chromosomal microarray analysis (CMA) and muation screening of *NDE1* gene were performed in an 8-month patient with severe congenital microcephaly, and/or his parents. Genetic studies found a 16p13.11 deletion containing *NDE1* gene, and a novel *NDE1* mutation c.555_556GC > CT on the non-deleted homolog, inherited from his phenotypically normal parents, respectively. The 2 bp nucleotide change results in a missense mutation p.K185 N and a nonsense mutation p.Q186X. We also conducted literaturte review to compare the clinical phenotypes of our patient to those of cases previously reported with *NDE1* mutations, and found all patients had mental retardation, severe microcephaly, and corpus callosum agenesis.

**Conclusion:**

This is the first Chinese reported with microcephaly caused by NDE1 mutations. NDE1 is a critical pathogenetic gene in severe congenital microcephaly. Sequencing NDE1 and CMA in patients with severe congenital microcephaly may be warranted.

## Background

The 16p13.11 locus is a genomic hotspot especially rich in low-copy repeats. These highly homologous DNA sequences are more likely to form copy number mutations through non-allelic homologous recombination [1]. The deletions of 16p13.11 are associated with a wide spectrum of disorders including schizophrenia, autism, mental retardation, intellectual disability, epilepsy, and mild microcephaly [2–6]. The 16p13.11 region contains a core set of genes including *NDE1*, the most important candidate gene for the neurodevelopmental phenotypes associated with the 16p13.11 microdeletions. *NDE1* encodes the nuclear distribution factor E-homolog 1 (nudE) protein that localizes to the centrosome and mitotic spindle poles, interacting with the cytoplasmic dynein complex and Lissencephaly-1 (LIS1) [7, 8]. This protein plays a crucial role in microtubule organization, mitosis and neuronal migration, essential for the process of mammalian encephalisation and human cerebral cortex growth [9].

To date, several cases of autosomal recessive extreme microcephaly caused by *NDE1* mutations have been reported. Herein we report that a patient, with severe congenital microcephaly, had 16p13.11 deletion containing *NDE1* gene combined with a novel *NDE1* mutation on the non-deleted homolog. We also compared the clinical phenotypes of all patients so far reported with *NDE1* mutations.

## Case presentation

**Fig. 1:**
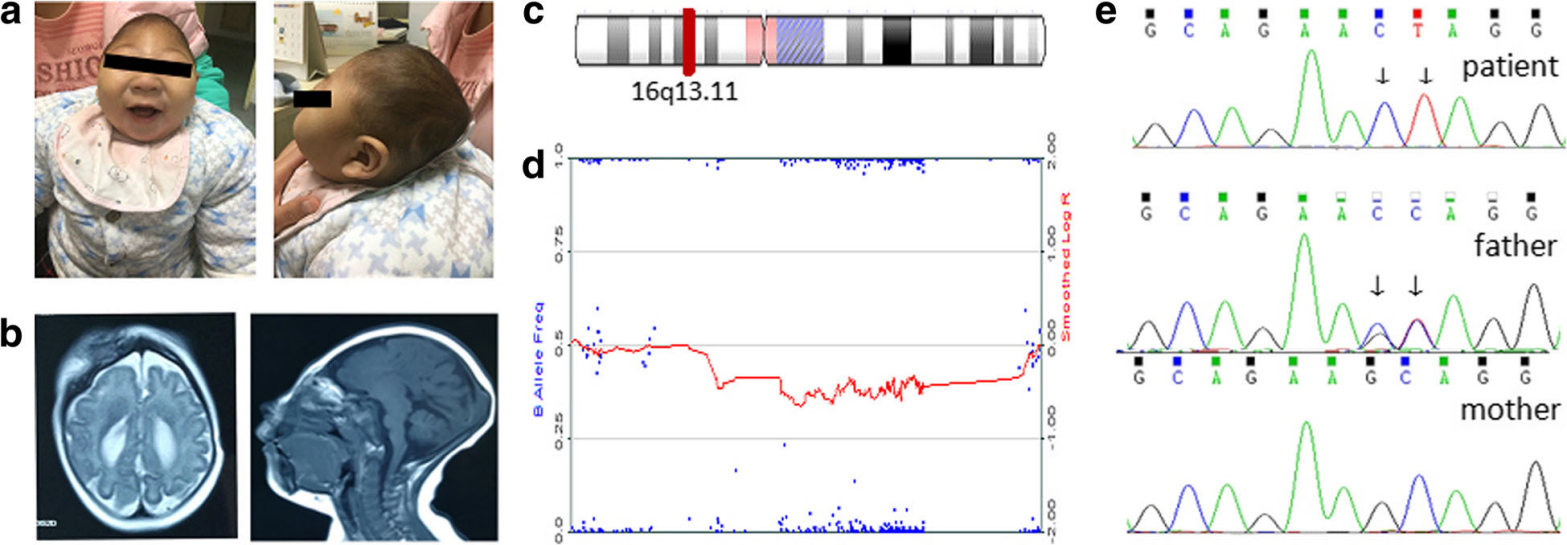
The typical phenotype and laboratory results. The patient at 8 months of age showed severe microcephaly (**a**). MRI showed the agenesis of the corpus callosum, enlargement of the posterior lateral ventricles, and a simplified gyral pattern of the cerebral cortex (**b**). The Diagram of chromosome 16 and the 1.22 M microdeletion on 16p13.11 in the patient detected by CMA (**c** and **d**). The *NDE1* sequence chromatogram of the patient and his parents, and arrows show the 2 base-pairs changes (**e**)

The CARE guidelines were followed in this case. The patient is an 8 months old male that had been diagnosed with extreme microcephaly. The patient was the first child of healthy, non-consanguineous parents, delivered after a 37 weeks gestation by cesarean section. The mother had a spontaneous abortion history after a 12 weeks gestation. The patient displayed microcephaly at birth but the physical parameters were not available. At the age of 8 months, physical examination showed severe microcephaly (Fig. 1a) with head circumference was 32 cm, 9.6 SD below the mean, marked hypertonia on his extremities, and global developmental delay. Magnetic resonance imaging scans showed the agenesis of the corpus callosum, enlargement of the posterior lateral ventricles, and a simplified gyral pattern of the cerebral cortex with scarce and wide gyri in parietal and occipital lobes (Fig. 1b). Electroencephalography showed slow background activity without epileptiform discharge.

Informed consent was obtained from the parents and the study protocols were reviewed and approved by the medical ethics committee of Wuhan Children’s Hospital, Tongji Medical College, Huazhong University of Science & Technology. Genomic DNA was extracted from peripheral blood using E.Z.N.A. ® MicroElute Genomic DNA Kit (Omega Bio-tek) from the patient and his parents. The samples from the patient and his parents were studied by the InfiniumOmniZhongHua-8 DNA Analysis BeadChip (Illumina), using for CNVs analysis larger than 30 Kb. CMA found that the patient had a 1.22 Mb deletion (hg19:15,070,916–16,292,323) on 16p13.11 inherited from his phenotypically normal mother (Fig. 1d). The deleted region contains 20 genes, including *ABCC1*, *ABCC6*, *FOPNL*, *MPV17L*, *MYH11*, *NDE1* and *NTAN1*. This boy had severe congenital microcephaly resembling that described in those patients with homozygous mutations in *NDE1* gene [9–12], so we attempted to detect genetic mutations in *NDE1* by performing Sanger Sequencing. The *NDE1* coding exons and flanking intronic sequences of the samples were amplified by PCR and then sequenced by Sanger sequencing using standard methods. Sequences were compared with normal reference sequences for *NDE1* (NCBI reference number NM_001143979.1, NP_001137451.1). The analysis identified a c.555_556GC > CT mutation in *NDE1* on the non-deleted homolog, inherited from his phenotypically normal father (Fig. 1e). The 2 bp nucleotide change results in a missense mutation (p.K185 N) and a stop codon (p.Q186X).

## Discussion and conclusion

We reported an 8-month patient with severe microcephaly, who harbored maternal 16p13.11 deletion combined with a paternal *NDE1* mutation. To date, 14 patients from 8 families with microcephaly involved *NDE1* gene have been reported, and these patients manifested severe congenital microcephaly, profound intellectual disability, and early onset seizure [9–12].

Microcephaly is a cortical malformation disorder characterized by severe impairment in brain development, small brain size, and a substantial reduction in neuronal number. Several genes have been identified to cause this condition, such as *ASPM*, *WDR62*, *CDK5RAP2*, *CENPJ*, *SIL*, and *BRIT1* [13–15], and these genes are involved in proliferation of the progenitor cells by affecting centrosome, mitotic spindle assembly, and cell cycle progression. NDE1-associated microcephaly is much more severe than the forms caused by defects in these genes, and patients with *NDE1* mutations often exhibit neuronal lamination defects, such as a simplified gyral structure or lissencephaly [9–12], and *NDE1* might have multiple roles in neocortical development.

Table 1 showed a comparison of the clinical findings of our patient with those cases reported previously [9–12]. All patients had mental retardation, severe microcephaly, and corpus callosum agenesis. Ventricular enlargement was presented in all patients except that Paciorkowski et al. did not describe this issue in their study. All but our patient displayed hypoplastic cerebellum or a proportionate reduction in the size of cerebellum, and epilepsy occurred in most of patients. Currently, it is unclear why the cerebellum of our patient normally appeared in brain MRI. These two mutations in our case, being inherited from their healthy heterozygous parents, were predicted to produce truncated proteins or deleted allele. As previously reported, immunoblot assay did not show detectable NDE1 protein in patient lymphoblast and, whereas the heterozygous parents showed ~50% reduction in protein level, suggesting the instability of mutant protein and subsequent degradation [10].

**Table 1:** A comparison of clinical features of patients with NDE1 deletion and/or mutations

	Bakircioglu et al.			Alkuraya et al.		Guven et al.	Paciorkowski et al.		Present work
Characteristics	F1 (P1-P4)	F2 (P5)	F3 (P6)	F4 (P7-P8)	F5 (P9-P10)	F6 (P11-P12)	P13	P14	P15
Ethnicity	Pakistani	Pakistani	Turkish	Saudi Arabian	Saudi Arabian	Slovak	Caucasian	Caucasian	Chinese
Number of patients	3 males & 1 female		1 male	1 male	2 females	1 male & 1 female	1 male & 1 female	1 male	1 male
Survival and reported age	died in the first 5 years of life		alive,NA	alive,7 years, 5.5 years	alive,3.5 years, NA	alive,19 years,17 years	alive,15 years	died at the age of 10 years	alive,8 months
Postnatal growth delay	–	–	–	+	+	+	+	+	+
Mental retardation	+	+	+	+	+	+	+	+	+
Microcephaly	+	+	+	+	+	+	+	+	+
Corpus callosum Agenesis	+	+	+	+	+	+	+	+	+
Ventricular enlargement	+	+	+	+	+	+	NA	NA	+
Cerebellum	Hypoplastic	Hypoplastic	Hypoplastic	Proportionate reduction	Proportionate reduction	Hypoplastic	Proportionate reduction	NA	normal
Seizures	+	+	+	–	+	+	+	single seizure during infancy	–
Muscle tone	NA	NA	NA	increased	decreased	NA	NA	NA	increased
prominent broad nasal bridge	NA	NA	NA	+	+	+	NA	NA	+
Genetic mutations	c.684_685delAC (hm)	c.684_685delAC (hm)	c.83 + 1G > T (hm)	c.684_685delAC (hm)	c.733dupC (hm)	c.-43_ + 83del (hm)	c.130C > T 16p13.11 deletion	c.908_909delGA 16p13.11 deletion	c.555_556GC > CT 16p13.11 deletion
Amino acid change	P229WfsX85	P229WfsX85	A29QfsX114	P229WfsX85	L245PfsX70	null allele	R44X	R303TfsX11	K185N&Q186X

*F* family, *P* patient; +, present; −, absent; *NA* not announced, *Hm* homozygotes

**Fig. 2:**
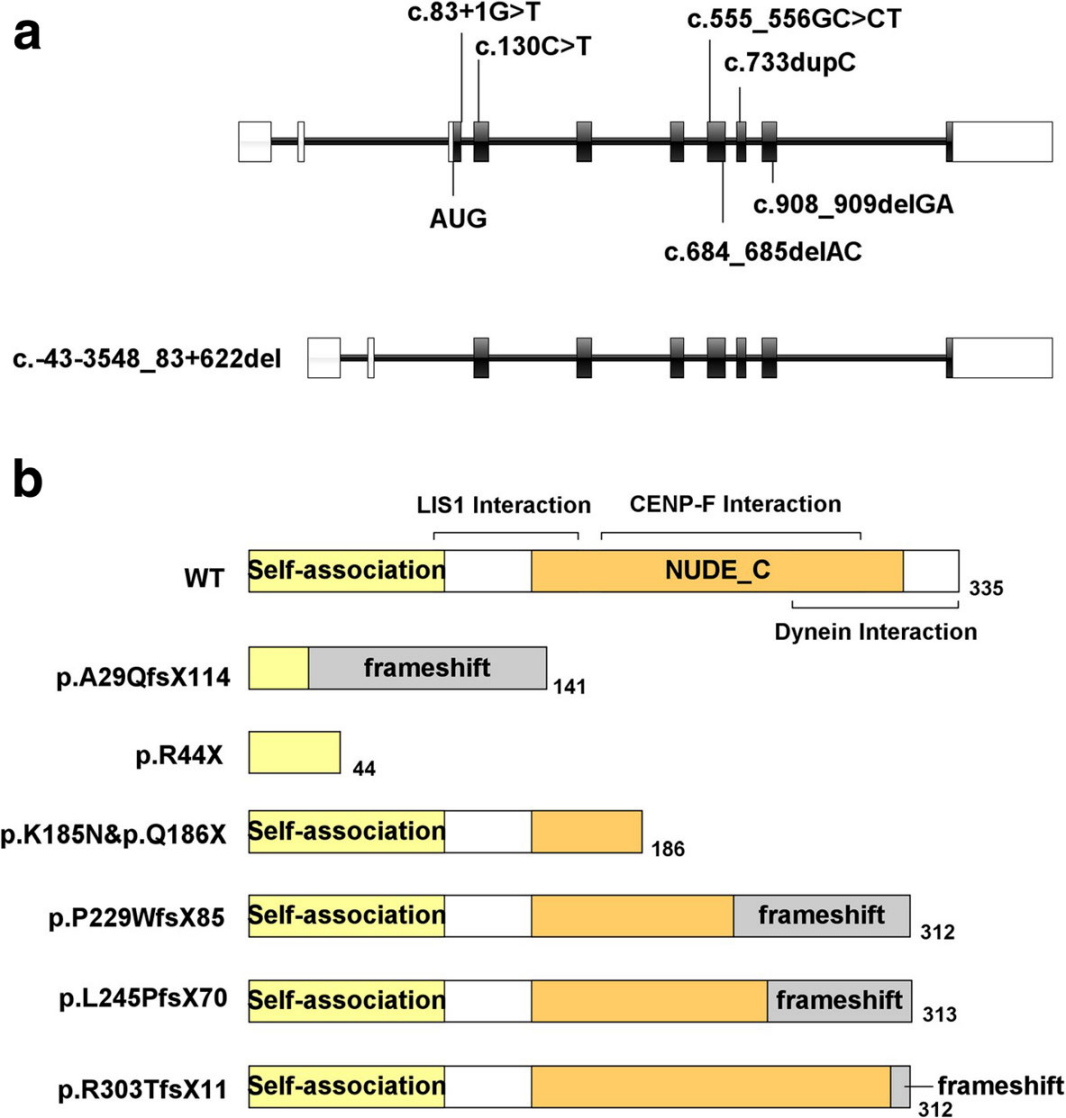
The diagram of human *NDE1* (NM_001143979.1). The coding sequences are shown in black and all the reported mutations are labeled (**a**). The predicted NDE1 protein derived from the mutations (**b**)

*NDE1* consists of 10 exons and a consensus start codon is located within exon 3 (NM_001143979.1) (Fig. 2a). Human NDE1 is a protein with 335 amino acids, harboring self-association domain, LIS1 interaction domain, and the C-terminal conserved domain (Fig. 2b). The C-terminal domain is essential for centrosomal localization of NDE1 and for its interaction with CENP-F (centromere protein F) and dynein [16–18]. LIS1 is essential for neural progenitor division and cortical migration, and its genetic mutations cause Miller-Dieker syndrome [19, 20]. CENP-F, a nuclear matrix component localizing at kinetochores during mitosis, is required for kinetochore localization of NDE1, LIS1, and Dynein [18]. Cytoplasmic dynein is a multisubunit complex involved in a variety of cellular functions, including mitotic spindle control, checkpoint regulation, transport of organelles, and directed nuclear and cell migration [16, 17, 21]. The corrected localization and activation of the dynein complex require the presence of many adaptors, such as CENP-F, LIS1, and NDE1. All these proteins work together to link spindle microtubes to kinetochores, being responsible for mitotic cell division. Recent studies have found that NDE1 is required in cell cycle progression and postmitotic neuronal migration, and NDE1 inhibition caused substantial numbers of radial glia progenitor cells’ nuclei to become arrested by impairing primary cilium dynamics, apical nuclear migration, and entry into mitosis [22]. All these findings could explain the defects in NDE1 contribute to extreme micrpcephaly and other neurodevelopmental conditions.

To date, several mutations in NDE1 have been reported. Bakircioglu et al. and Alkuraya et al. reported three distinct homozygous frameshift mutations of NDE1, c.83 + 1G > T (p.A29QfsX114), c.684_685delAC (p.P229WfsX85) and c.733dupC (p.L245PfsX70) [9, 10]. The c.83 + 1G > T leads to the formation of a novel splice site and to a frameshift after NDE1 exon 3, and the novel open reading frame would be predicted to maintain the N-terminal 28 native amino acids followed 113 novel amino acids [9]. The mutant protein completely lost the conserved C-terminal domain, including interacting domains with CENP-F and dynein, and the centrosomal localization domain, the majority of LIS1 binding domain as well [9]. The c.684_685delAC resulted in a premature stop codon at position 313, and the truncated protein lost the highly conserved C-terminal CENP-F and dynein interaction domain, and also failed to present at the centrosome. However, the mutant protein would be predicted to interact with LIS1 [9]. The c.733dupC resulted in a truncated protein with 244 native amino acids of the wild-type protein on the N terminus followed by 69 abnormal amino acids. The consequence of this mutation is similar to that of c.684_685delAC, which have been proved in vitro studies [9, 10], and these truncated mutant proteins failed to bind to the dynein complex and to localize properly to the centrosome. Guven et al. identified a homozygous deletion that encompasses NDE1 exon 3 containing the initiation codon, predicted to result in a null allele [11]. Paciorkowski et al. describe two patients with inherited deletions of 16p13.11 encompassing the entire NDE1 gene on one allele combined with a nonsense mutation (p.R44X) or a frameshift mutation (p.R303TfsX11) in the non-deleted NDE1 [12]. Our patient had the deletion of the entire NDE1 gene on one allele combined with a c.555_556GC > CT on the non-deleted homolog, resulting in a missense mutation (p.K185 N) and a nonsense mutation (p.Q186X). The nonsense mutation is predicted to cause protein truncation in the C-terminal domain of NDE1. If stable, the truncated protein may fail to localize properly to the centrosome due to the loss of domains interacting with CENP-F and dynein. This heterozygote of c.555G > C (p.K185 N) and c.556C > T was found to be only presented in one case in East Asian among in populations with more than 120,000 individuls, including East asian, African, Latino, European, etc. in genome aggregation database (http://gnomad.broadinstitute.org/), respectively. There was no corresponding literature about these two variants, and it is unclear whether they were presented in one person. There is no report in the first Asian Diploid genome (http://yh.genomics.org.cn/).

In, conclusion, we reported a NDE1 genetic mutation combined with 16q13.11 microdeletion in a boy with extreme microcephaly. To our knowledge, this is the first Chinese reported with microcephaly caused by NDE1 mutations. Our findings enrich the understanding of NDE1 mutations and the associated phenotypes. It is warranted to perform mutation screening in NDE1 gene for patients with severe micropcephaly.

## Nomenclature

### Nucleotide symbol

- *A* adenosine
- *C* cytidine
- *G* guanosine
- *T* thymidine
- *U* uridine

### Amino acid residue symbol

- *A* Alanine
- *K* Lysine
- *L* Leucine
- *N* Asparagine
- *P* Proline
- *Q* Glutamine
- *R* Arginine
- *T* Threonine
- *W* Tryptophan
- *X* stop codon

## Abbreviations

ABCC1: ATP binding cassette subfamily C member 1
ABCC6: ATP binding cassette subfamily C member 6
ASPM: abnormal spindle microtubule assembly
bp: base pairs
BRIT1: BRCA1 C terminus repeat inhibitor of hTERT
CDK5RAP2: CDK5 regulatory subunit associated protein 2
CENP-F: centromere protein F
CENPJ: centromere protein J
CMA: Chromosomal Microarray Analysis
CNVs: copy number variations
del: deletion
dup: duplication
FOPNL: FGFR1OP N-terminal like
fs: frameshift
Kb: kilobase(s) or 1000 bp
LIS1: Lissencephaly-1
MPV17L: MPV17 mitochondrial inner membrane protein like
MYH11: myosin heavy chain 11
NDE1: nuclear distribution factor E-homolog 1
NTAN1: N-terminal asparagine amidase
PCR: Polymerase Chain Reaction
SD: standard deviation
SIL: STIL, centriolar assembly protein
WDR62: WD repeat domain 62
